# ConvNeXt steel slag sand substitution rate detection method incorporating attention mechanism

**DOI:** 10.1038/s41598-023-37676-y

**Published:** 2023-06-30

**Authors:** Shengjie Teng, Lin Zhu, Yunze Li, Xinnian Wang, Qiang Jin

**Affiliations:** 1 College of Hydraulic and Civil Engineering, Xinjiang Agricultural University, Ürümqi, 830052 China; 2 Department of Xinjiang Corps Urban Construction Group Co, Ürümqi, 830052 China

**Keywords:** Civil engineering, Information technology

## Abstract

The proportion of natural sand replaced by steel slag sand affects the volumetric stability of steel slag mortar and steel slag concrete. However, the steel slag substitution rate detection method is inefficient and lacks representative sampling. Therefore, a deep learning-based steel slag sand substitution rate detection method is proposed. The technique adds a squeeze and excitation (SE) attention mechanism to the ConvNeXt model to improve the model's efficiency in extracting the color features of steel slag sand mix. Meanwhile, the model's accuracy is further enhanced by using the migration learning method. The experimental results show that SE can effectively help ConvNeXt acquire images' color features. The model's accuracy in predicting the replacement rate of steel slag sand is 87.99%, which is better than the original ConvNeXt network and other standard convolutional neural networks. After using the migration learning training method, the model predicts the steel slag sand substitution rate with 92.64% accuracy, improving accuracy by 4.65%. The SE attention mechanism and the migration learning training method can help the model acquire the critical features of the image better and effectively improve the model's accuracy. The method proposed in this paper can identify the steel slag sand substitution rate quickly and accurately and can be used for the detection of the steel slag sand substitution rate.

## Introduction

With the continuous development of the construction industry, the demand for natural sand is increasing. However, large-scale use of natural sand tends to cause environmental depletion, resulting in a shortage of sand resources and soaring prices, so there is a need to find substitutes for natural sand^1,2^. Steel slag is an industrial waste product produced in the steelmaking process, which is inexpensive, has a hard texture and good wear resistance, and can be used as a substitute for natural sand^3–5^. However, steel slag sand contains harmful components such as free calcium and magnesium oxide. When the replacement ratio is too high, it will affect the workability and volumetric stability of steel slag mortar and steel slag concrete^6^. Jiangfan^7^ showed that when steel slag sand is mixed with more than 60%, the problem of failed stability may occur. Rehman and Panetal^8,9^ proposed that steel slag cannot replace more than 60% of fine aggregates, and the fluidity of fresh concrete decreases with the increase of the replacement ratio. By studying the effect of steel slag sand replacement rate on mortar, Mao Feikai^10^ found that the optimum replacement rate was 20%, and the maximum replacement rate was 60% for equal volume replacement. Reasonable control is needed when using steel slag sand as a natural substitute. Therefore, there is an urgent need for a fast and accurate steel slag sand substitution rate testing method to control steel slag sand better, thus reducing engineering risks and improving steel slag sand utilization.

With the development of computer vision, image processing technology is widely used in building materials. Ma^11^ used digital image processing technology to describe the morphological characteristics of coarse aggregate, including angle, sphericity, texture, and fractal dimension. The difference in morphological characteristics between recycled coarse aggregate and natural gravel coarse aggregate was discussed. Han^12^ evaluated the characteristics and distribution of coarse aggregates based on two-dimensional images of concrete cross-section images. Cao^13^ proposed a graham algorithm for complex image convex hull processing to evaluate the shape characteristics of the measured aggregate particles quickly. Traditional image processing technology has achieved specific recognition results in the direction of building materials. However, it relies on manual feature extraction and complex parameter adjustment process, and the technical threshold is high.

With the rise of deep learning, the convolutional neural network is superior to traditional image processing technology in computer vision. It has good learning and generalization ability and does not need to extract features manually. After AlexNet^14^ won the ImageNet Visual Recognition Challenge in 2012, the convolutional neural network model developed rapidly. Representative models include VGGNet^15^, ResNet^16^, MobileNet^17^, ShuffleNet^18^, and Efficient^19^. Dan^20^ uses a convolutional neural network (U-NET++) to segment aggregated particles, which can accurately obtain the geometry of the particles appearing on the side of the asphalt mixture. An identification and analysis method of asphalt mixture segregation and weakening is proposed. Jean^21,22^ uses a convolutional neural network (CNN) to analyze the recycled aggregate image and give the composition of recycled aggregate in real-time. Wenjun Wang^23^ focuses on the segmentation of aggregates in concrete settlement images. An extrusion and excitation module was added to the model ResNeXt 50. Adaptively recalibrate the channel feature response to improve the efficiency of feature extraction. A concrete aggregate segmentation method based on deep learning is proposed. The convolutional neural network can classify complex images, and the structure is simple and applicable, which can better detect the substitution rate of steel slag sand.

Based on deep learning technology, this paper proposes a steel slag sand image classification model SE-ConvNeXt based on a convolutional neural network. The classification model adds a channel attention mechanism SE^24^ (squeeze and excitation network) to ConvNeXt^25^ for the characteristics of the color change of steel slag mixed sand. The ability of ConvNeXt to obtain the color features of steel slag mixed sand images is enhanced, and the accuracy of ConvNeXt in the classification task of steel slag sand substitution rate is improved. On this basis, the transfer learning^26^ method is used to train the network model to improve the model's accuracy further to identify the steel slag mixed sand image.

The main contributions of this paper are as follows:

1. To identify the substitution rate of steel slag in different kinds of mixed sand, three kinds of mixed sand samples were made by mixing steel slag sand with three different kinds of sand. It helps the model to thoroughly learn the characteristics of different types of steel slag mixed sand so that the network can adapt to the detection of different mixed sand.
2. Aiming at the problem of image difference of steel slag mixed sand: the SE attention mechanism module is added before the ConvNeXt module. Help the model use the image's practical information better to learn and identify the target and improve the efficiency and accuracy of the model.
3. To address the issue of lower accuracy in identifying the steel slag substitution rate, we employed transfer learning to train the network. Firstly, we selected a dataset of mixed sand samples to pre-train the SE-ConvNeXt model and saved the weights of the pre-trained model. Secondly, we transferred all the consequences from the pre-trained model to the SE-ConvNeXt network. Finally, we train the model using three sets of mixed sand samples, updating all the parameters. This approach significantly improved the learning efficiency of the model and resulted in higher accuracy.

## Materials and methods

In order to complete the classification recognition of steel slag sand substitution rate, this paper firstly made mixed sand samples according to the steel slag sand substitution rate, and obtained mixed sand images by professional camera to divide the data set. Finally the dataset is used for training, validation and testing of the network to obtain the results.

### Mixed sand dataset

The production of the mixed sand dataset in this paper includes the following four steps.

**Figure 1:**
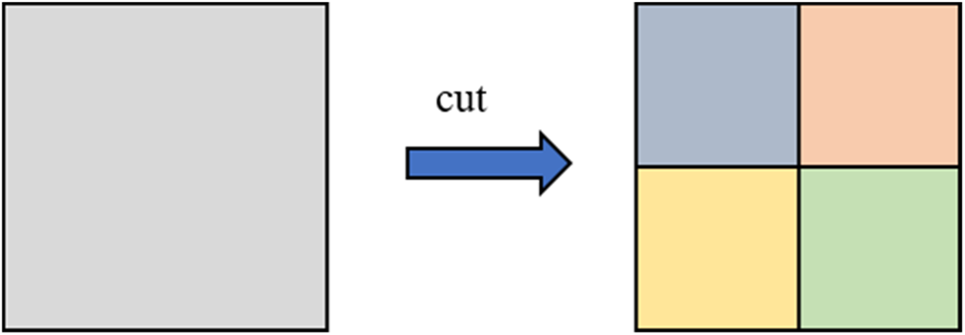
Cutting method.

1. Composition of samples. Standard sand and two kinds of natural sand with different grain sets were selected to be mixed with steel slag sand, respectively, to make samples of three kinds of mixed sand. A is the mixed sample of standard sand (medium sand) and steel slag sand (medium sand), B is the mixed sample of natural sand (coarse sand) and steel slag sand, and C is the mixed sample of natural sand (medium sand) and steel slag sand.
2. Production of mixed sand. Firstly, the samples were taken by GB/t14684-2022 "Sand for Construction." Next, according to the above configuration of mixed samples, the corresponding mixed samples were made according to the steel slag sand substitution rate of 0, 20, 40, 60, 80, and 100%. Finally, the mixed samples were placed into the blender separately, shaken, and stirred for 60 s to mix thoroughly and reduce the experimental error caused by uneven mixing. The fineness modulus was used to classify mixed sand's coarse and fine grades. Among the three mixed sand samples, the A and C mixed sand samples were medium sand, the B mixed sand samples with 0–40% substitution were coarse sand, and more than 60% was medium sand.
3. Mixed sand image acquisition. The professional camera was placed 10 cm directly above the mixed sand, and the specific parameters of the camera are shown in Table 1. One hundred images were taken for each of the blended sands of each substitution rate. A list of typical images was taken, as shown in Table 2.
4. Production of the dataset. Each acquired image was cropped into four copies on average. The cropping method is shown in Fig. 1; after processing, there are 400 images of mixed sand for each substitution rate and 7200 images in total for the data set of three sets of mixed sand. The images of the three datasets were compressed to 224 × 224 pixels, and the dataset labels were made according to the steel slag sand substitution rate. Each group is divided into data sets according to the percentage of 60% of the training set, 20% of the validation set, and 20% of the test set.

**Table 1:** Camera parameters.

	Photosensibility (iso)	Shutter (s)	Automatic focus (af)	White balance (k)	Pixels (pt)
Parameters	50	1/15	AF-C	4200	2736 × 2736

**Table 2:**
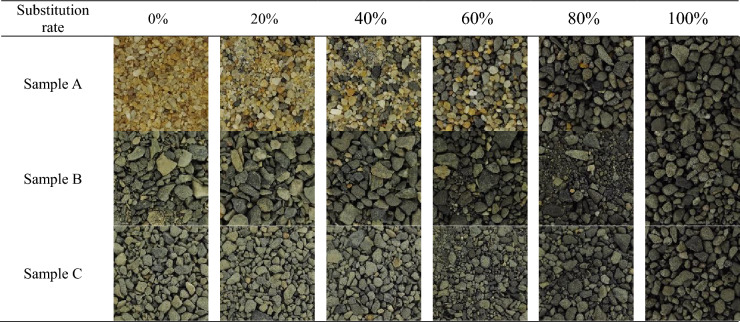
Typical images of each substitution rate of steel slag mixed sand.

### Models

#### ConvNeXt model

The SE-ConvNeXt network architected in this paper uses the ConvNeXt network as the main structure. The ConvNeXt network is a convolutional neural network model based on the ResNet structure borrowed from the Swin Transformer^27^ architectural strategy. ConvNeXt offers better performance and a more straightforward model structure than Swin Transformer. Details of the ConvNeXt network are shown in Table 3.

**Table 3:** Network structure information.

Structure	Input size	Convolution kernel, channel, stride(s)	Output size
Patchify	224 × 224 × 3	4 × 4, 96, s4	56 × 56 × 96
Downsample	56 × 56 × 96	2 × 2, 96, s2	56 × 56 × 96
ConvNeXt block	56 × 56 × 96	$$\left[\begin{array}{c}{\text{d}}{7}\times {7}\text{,} \, {96}\text{,} \, {\text{s}}{1}\\ {1}\times {1}\text{,} \, {384}\text{,} \, {\text{s}}{1}\\ {1}\times {1}\text{,} \, {96}\text{,} \, {\text{s}}{1}\end{array}\right]\times {3}$$	28 × 28 × 192
Downsample	28 × 28 × 192	2 × 2, 192, s2	28 × 28 × 192
ConvNeXt block	28 × 28 × 192	$$\left[\begin{array}{c}{\text{d}}{7}\times {7}\text{,} \, {192}\text{,} \, {\text{s}}{1}\\ {1}\times {1}\text{,} \, {768}\text{,} \, {\text{s}}{1}\\ {1}\times {1}\text{,} \, {192}\text{,} \, {\text{s}}{1}\end{array}\right]\times {3}$$	14 × 14 × 384
Downsample	14 × 14 × 384	2 × 2, 384, s2	14 × 14 × 384
ConvNeXt block	14 × 14 × 384	$$\left[\begin{array}{c}{\text{d}}{7}\times {7}\text{,} \, {384}\text{,} \, {\text{s}}{1}\\ {1}\times {1}\text{,} \, {1536}\text{,} \, {\text{s}}{1}\\ {1}\times {1}\text{,} \, {384}\text{,} \, {\text{s}}{1}\end{array}\right]\times {9}$$	7 × 7 × 768
Downsample	7 × 7 × 768	2 × 2, 768, s2	7 × 7 × 768
ConvNeXt block	7 × 7 × 768	$$\left[\begin{array}{c}{\text{d}}{7}\times {7}\text{,} \, {768}\text{,} \, {\text{s}}{1}\\ {1}\times {1}\text{,} \, {3072}\text{,} \, {\text{s}}{1}\\ {1}\times {1}\text{,} \, {768}\text{,} \, {\text{s}}{1}\end{array}\right]\times {3}$$	7 × 7 × 768

This is a note. Patchify layer denotes the initial downsampling layer. Downsample layer denotes the downsampling layer between the two ConvNeXt Blocks. d7 × 7, 96, s1 denotes a deep convolutional layer of size 7 × 7, with 96 channels and a step size of 1. 4 × 4, 96, s4 denotes a convolutional layer of size 4 × 4, with 96 channels and a step size of 4.

**Figure 2:**
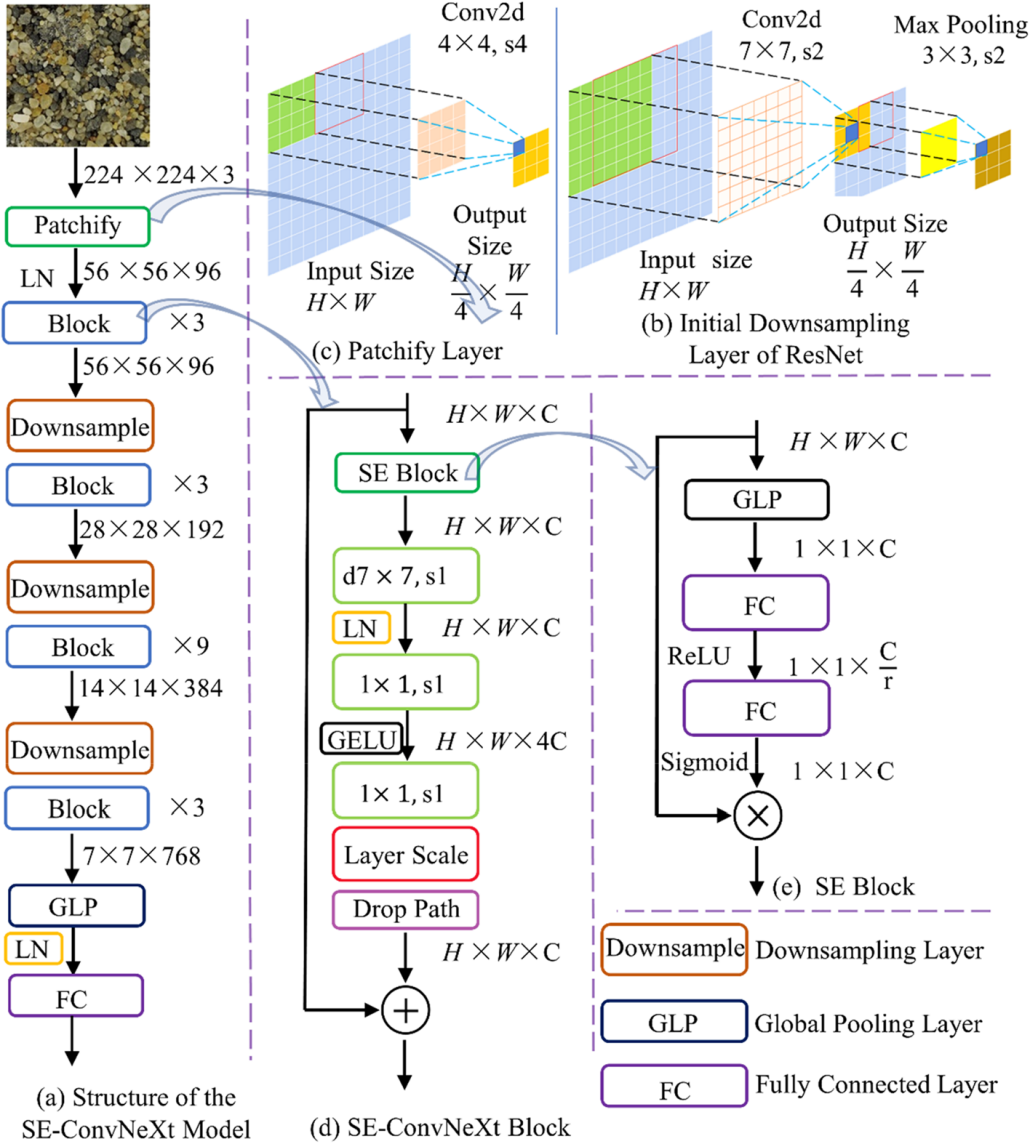
SE-ConvNeXt model: (**a**) structure of the SE-ConvNeXt model, (**b**) patchify layer, (**c**) initial downsampling layer of ResNet, (**d**) SE-ConvNeXt block, (**e**) SE block.

ConvNeXt comprises three parts: downsampling layer, ConvNeXt block, and fully connected layer. The downsampling layer of ConvNeXt includes the patchy layer (the initial downsampling layer), the downsampling layer between ConvNeXt blocks, and the downsampling layer between ConvNeXt block and the fully connected layer. ConvNeXt's patchy layer borrows the architectural approach of Swin Tansformer's patchy layer. The patchy layer is constructed with a convolutional layer of size 4 × 4 and stride four instead of using the traditional structure of downsampling sublayer in ResNet. As the patchy layer in Fig. 2b and c; b is the downsampling layer of ResNet and c is the patchy layer of the ConvNeXt network. The downsampling layer between the two ConvNeXt blocks uses a convolutional layer of size 2 × 2 and a stride of 2. The downsampling layer between the ConvNeXt block and the fully connected layer is composed using a global average pooling layer.

The stacking number of Conv Ne Xt blocks imitates the stacking ratio of Stages in the Swin Transformer. It was adjusted from (3,4,6,3) to (3,3,9,3), as shown in Table 3. The ConvNeXt block uses the inverted bottleneck in the Swin Transformer stage, as shown in the SE-ConvNeXt block module diagram in Fig. 2a. First, a 7 × 7 deep convolutional layer and two 1 × 1 convolutional layers are used to extract the features of the input image. Next, the Layer Scale^28^ operation is performed to scale the data for each channel. Finally, a DropPath^29^ layer is added to deactivate the main branch structure of the model randomly in order to prevent the phenomenon of overfitting and improve the generalization ability of the model.

The fully-connected layer is located at the end of the entire network. The fully-connected layer compresses the features extracted from the convolutional and pooling layers and completes the classification function of the model based on the compressed features.

ConvNeXt combines the strategies of the Swin Tansformer model architecture in its overall architecture and focuses on the differences between the ResNet model and the details of the Swin Tansformer model. The GELU (Gaussian Error Linear Units)^30^ activation function is commonly used in Transformer models, while the ReLU (Rectified Linear Units)^31^ activation function is commonly used in ResNet models. Transformer uses fewer activation functions instead of connecting an activation function to each convolutional or fully connected layer. Similarly, Transformer uses fewer data normalization operations, using LN (Laryer Normalization) rather than BN (Batch Normalization), commonly used in ResNet models. ConvNeXt draws on the strategy of the Swin Transformer model architecture by following the GELU activation function after the first 1 × 1 convolutional layer in the ConvNeXt block and using the LN after 7 × 7 deep convolution as the SE-ConvNeXt block module in Fig. 2d.

#### SE-Net model

Table 2 shows that the mixed sand samples of A, B, and C are darker when the steel slag sand substitution rate is 100%, indicating that the pure steel slag sand is darker. At a low substitution rate, the color characteristics of the three sand blends differed significantly. As the substitution rate increases, the proportion of steel slag sand increases, and the color characteristics of the three sand blends gradually become similar. The color characteristics of blended sand images with similar substitution rates are similar in the same blended sand samples. According to the characteristics of the steel slag mixed sand samples, applying the SE block to the convolutional neural network should result in a more significant performance improvement with a slight increase in computation.

SE-Net: SE-Net is a network structure proposed by Jie Hu's team^24^, which used SE-Net and won the ImageNet 2017 competition for the image classification task. The process of an SE block is divided into two steps, Squeeze and Excitation: (1) Squeeze is the global compressed feature volume of the current feature layer by performing global average pooling on the feature layer; (2) The excitation is to get the weights of each channel in the feature layer by two fully connected bottleneck layers and use the weighted feature layer as the input of the next layer of the network. As shown in Fig. 2e for the SE block.

#### SE-ConvNeXt model

In Transformer, the attention mechanism module is located before the multi-layer perceptron layer, and this paper uses the SE block to emulate this architectural approach. The SE-ConvNeXt block is formed by placing the SE modules before the ConvNeXt block, as the SE-ConvNeXt block in Fig. 2d. Therefore, the main structure of the SE-ConvNeXt model is the same as that of the ConvNeXt model. As shown in the structure of the SE-ConvNeXt model in Fig. 2a.

### Transfer learning

Transfer learning is a machine learning method that improves the performance of a model on a target task by applying knowledge and experience learned in one domain to another related field. In transfer learning, previously known models or feature representations are used as a starting point for a new task, accelerating and enhancing the learning process. Through transfer learning, a model can leverage the acquired knowledge and patterns to adapt to the specific requirements of the new task, even when the training data for the target task is limited or not sufficiently representative. Transfer learning can be achieved by sharing lower-level features, adjusting network structures, tuning model parameters, and other approaches.

By selecting one set of mixed sand samples, we performed pretraining on SE-ConvNeXt and saved the weights of the pre-trained model. These pre-trained weights represent the learned parameter values of the model during the pretraining phase, capturing its understanding and representation of the mixed sand data. The trained consequences can include the weights of the model's convolutional layers, pooling layers, fully connected layers, and other components. By utilizing these pre-trained weights, we can leverage the knowledge already acquired on one set of mixed sand data and transfer it to the classification task on the three sets of diverse sand data, thereby accelerating and improving the training and performance of the model. To achieve the best experimental results, we transferred the pre-trained weights to all layers of SE-ConvNeXt and used all the model parameters in training on the three sets of mixed sand samples. This approach is particularly suitable for situations with similarities between the source and target tasks. The knowledge and features learned by the pre-trained model on the source task can be more easily transferred to the target task.

## Experiments and results

Experimental Device: SE-ConvNeXt is implemented in Keras deep learning framework based on CNN using python language.

SE-ConvNeXt Detection Process: First, we train the network using three validation sets and test sets and test the SE-ConvNeXt network model performance with the test sets. Next, the SE-ConvNeXt network is compared with the ConvNeXt network and other advanced networks to evaluate the advantages and disadvantages of the improved SE-ConvNeXt network. Finally, transfer learning is used to train SE-ConvNeXt and compare the model performance before and after transfer learning.

### Confusion matrix

In order to test the stability of the SE-ConvNeXt model, this paper trained five sets of SE-ConvNeXt models with five cross-validations using three data sets. The classification accuracies of the five groups of models on the test set were 87.64, 89.24, 87.22, 88.61, and 87.22%, respectively; the average classification accuracy was 87.99%, with a standard deviation of 0.902%. The above results show that SE-ConvNeXt has good stability.

**Figure 3:**
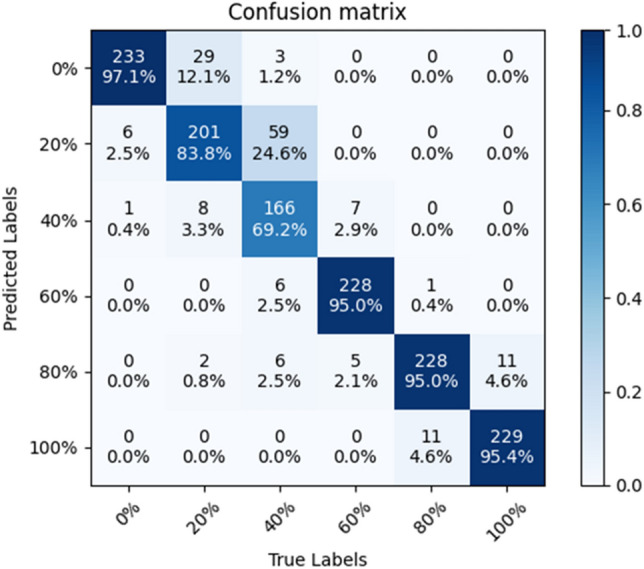
Confusion matrix of SE-ConvNeXt.

The model with 89.24% accuracy is used as an example to produce the confusion matrix shown in Fig. 3. The graph's horizontal axis represents the real mixed sand substitution rate, and the vertical axis represents the model-predicted mixed sand substitution rate. The diagonal line is the correct prediction result, and the darker the cube color, the more images are correctly predicted. Figure 3 shows that the model predicts four substitution rates of 0, 60, 80, and 100% with an image accuracy of more than 90%, while the accuracy of predicting a 40% substitution rate is only 69.2% and the accuracy of predicting 20% substitution rate is only 83.8%. Most of the images incorrectly predicted by the model were adjacent substitution rate images. The dataset of mixed sand is made according to the steel slag sand substitution rate, so the adjacent substitution rate images have approximate color characteristics. The confusion matrix shows the results of the confusion images, which conform to the regularity of the data set, and thus shows the reliability of the SE-ConvNeXt model for steel slag sand substitution rate detection.

In order to determine the reasons for the easy confusion of 20 and 40% substitution rate sand mixes, this paper examines the test sets of three sand mix samples, A, B, and C, respectively. Among them, SE-ConvNeXt predicted the test set of sample A with 96.88% accuracy, and the model predicted sample A with a high accuracy rate. There is a large difference in the color of standard sand and steel slag sand, so the model can easily distinguish the substitution rate of steel slag sand in sample A.

**Figure 4:**
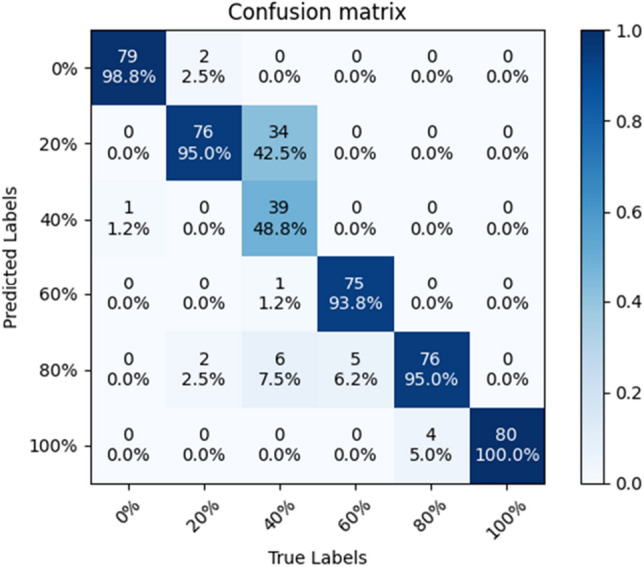
Confusion matrix of SE-ConvNeXt predicted B-sample test set.

**Figure 5:**
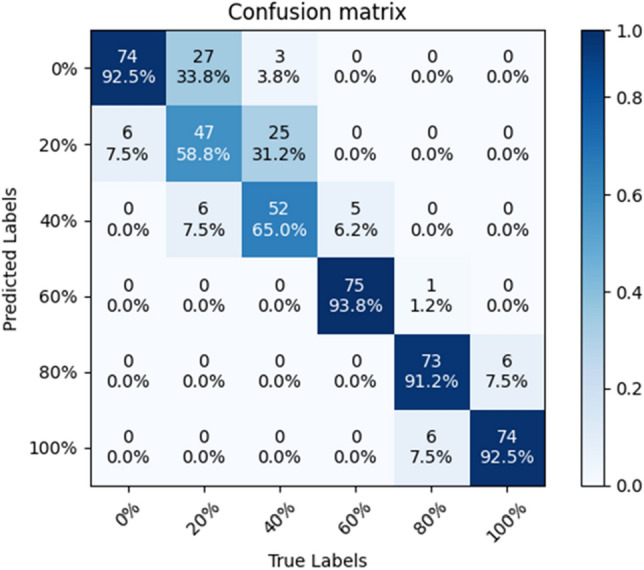
Confusion matrix of SE-ConvNeXt predicted C-sample test set.

The accuracy of SE-ConvNeXt in predicting the B sample test set was 88.54%, and the confusion matrix is shown in Fig. 4. The accuracy rate of 40% substitution was only 48.75%, and the predicted error images were mostly 20% substitution. The model predicted the C sample test set with 82.29% accuracy, and the confusion matrix is shown in Fig. 5. The accuracy rate of 40% substitution is only 65%, and the predicted error images are mostly 20% substitution. The accuracy of 20% substitution was only 58.75%, and the predicted error images were mostly 0% substitution. When the steel slag sand substitution rate is low, the steel slag sand accounts for less of the mixed sand, and less of the steel slag sand color characteristics are shown in the image. So the model needs to be more accurate in identifying sand mixes with 20 and 40% substitution rates. As the substitution rate of steel slag sand increases, the proportion of steel slag sand gradually increases, and the color characteristics of steel slag sand dominate. So the model has high accuracy in images where the steel slag sand substitution rate exceeds 60%. When the substitution rate of steel slag sand reaches 60%, the problem of inferior mortar stability will occur. The model can accurately predict the mixed sand with a 60% substitution rate or more, so the steel slag sand substitution rate can be detected by the SE-ConvNeXt model to reduce the problems such as failure of the stability.

### Ablation experiment

In order to verify the effect of the SE attention mechanism block on ConvNeXt, this paper compares ConvNeXt and SE-ConvNeXt. Similarly, five groups of ConvNeXt models were trained, and the accuracy of the five groups on the test set is 85.35, 86.32, 85.90, 86.81, and 86.88%, with an average accuracy of 86.25%. The five sets of the accuracy of ConvNeXt after adding the SE attention mechanism module were 87.64, 89.24, 87.22, 88.61, and 87.22%, with an average accuracy of 87.99%. SE-ConvNeXt improved the accuracy over ConvNeXt by 1.74%, while the parameters increased by only 5.84%. Therefore, in the steel slag sand substitution rate classification task, the SE attention mechanism can significantly improve the accuracy of the classification task with a slight increase in computation.

**Figure 6:**
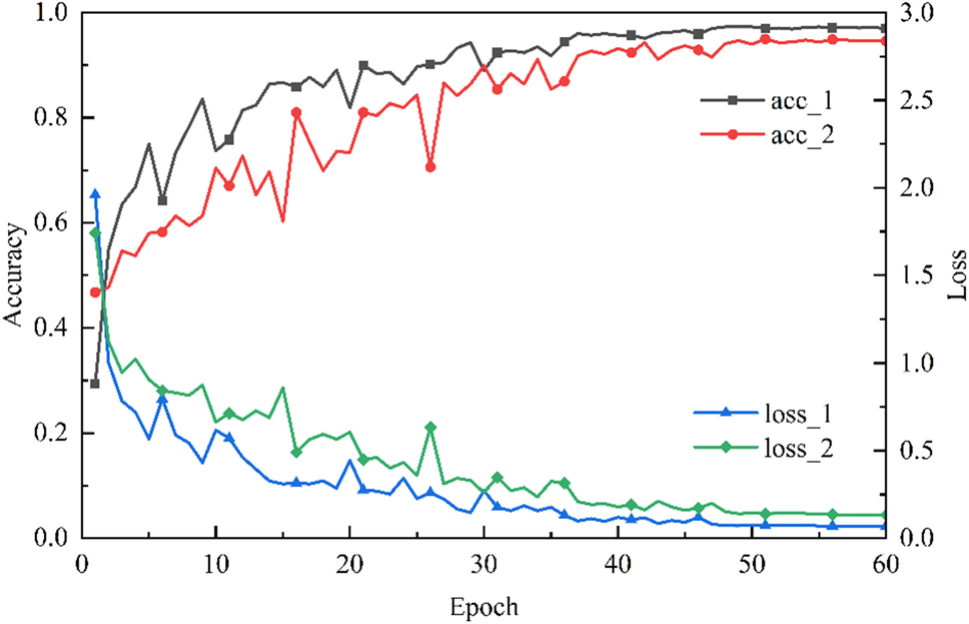
Comparison of accuracy and loss values between SE-ConvNeXt and ConvNeXt.

Figure 6 compares the accuracy and loss values of the two models, SE-ConvNeXt and ConvNeXt. acc_1 and loss_1 are the accuracy and loss values on the validation set during SE-ConvNeXt training. acc_2 and loss_2 are the accuracy and loss values on the validation set during ConvNeXt training. They compare the accuracy and loss values of the two models. In the early training period, the loss value and accuracy converge faster. The falling gradient of the loss value curve and the rising gradient of the accuracy curve of the SE-ConvNeXt model are much larger than those of ConvNeXt. As the model is gradually trained, the SE-ConvNeXt model converges much faster than ConvNeXt, with a smaller range of curve fluctuations. The SE-ConvNeXt accuracy reached 97.0%, and the loss value converged to 0.069. The ConvNeXt accuracy reached 94.6%, and the loss value converged to 0.132. Comparison of accuracy and loss values shows that the ConvNeXt model with the addition of SE has higher accuracy, lower loss values, a more stable training process, and better convergence.

**Figure 7:**
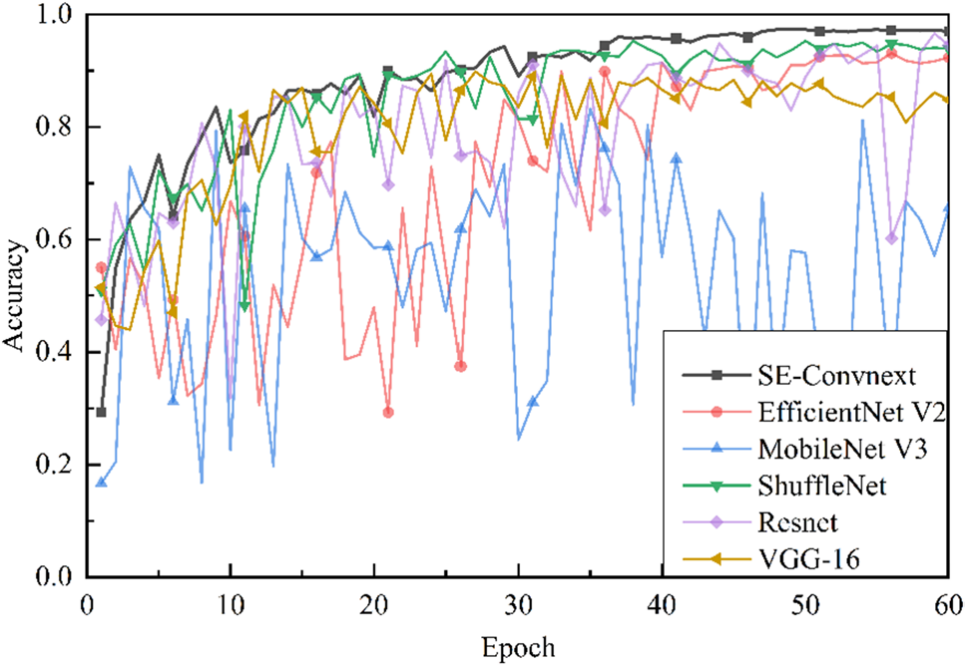
Accuracy of convolutional neural network training process on the validation set. This is a note. X-axis is the training epoch, Y-axis is the accuracy of the corresponding network on the validation dataset.

**Figure 8:**
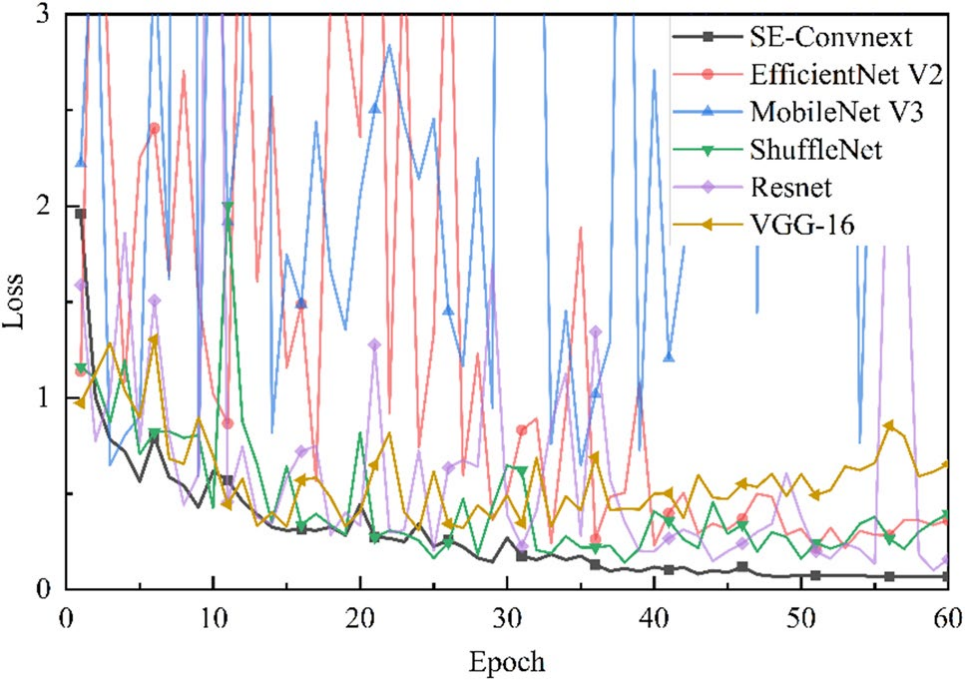
Loss values on the validation set during the training of the convolutional neural network. This is a note. X-axis is the training epoch, Y-axis is the loss of the corresponding network on the validation dataset.

Comparison of Convolutional Neural Networks. To further validate the effectiveness of the improved model in the steel slag sand substitution rate classification task, SE-ConvNeXt was compared with other convolutional neural networks. Five models EfficientNet V2^32^, MobileNet V3^33^, ShuffleNet V2^34^, VGG-16, and Resnet-50, were trained using the dataset. Figure 7 shows the accuracy of the validation set during the training of EfficientNet V2, MobileNet V3, ShuffleNet V2, VGG-16, Resnet-50, and SE-ConvNeXt. Figure 8 shows the loss values on the validation set while training the six convolutional neural networks mentioned above. According to the trend of accuracy and loss values shown in Figs. 7 and 8. MobileNet V3 and Resnet-50 need better training results. The EfficientNet V2 network converges slowly at the beginning, while the VGG-16, ShuffleNet V2, and SE-ConvNeXt networks converge faster at the beginning of training. As the model is gradually trained, the accuracy of EfficientNet V2 finally reached 92.4%, and the loss value converged to 0.363, with a wide range of curve fluctuations in the late training period. The VGG-16 accuracy reached 84.81%, and the loss value converged to 0.655. The curve fluctuated in a wide range in the late training period, with a downward trend in the accuracy curve and an upward trend in the loss value curve. The ShuffleNet V2 accuracy finally reached 93.9%, the loss value converged to 0.397, and the curve fluctuated less in the late training period. The absolute accuracy of SE-ConvNeXt is 97.0%, with a loss value of 0.069, and the curve fluctuates less in the late training period. In summary, SE-ConvNeXt has a faster convergence rate, higher accuracy, and stable training process in the steel slag sand substitution rate classification task compared with other models.

Table 4 shows the comparison of training parameters and accuracy of each model, and the validation of MobileNet V3 and Resnet-50 was not performed for these two networks because of their poor training results. The larger the parameters, the more computationally intensive the model is, comparing parameters and accuracy. The VGG-16 parameters are much larger than the rest of the network, and the accuracy is lower. The parameter values of SE-ConvNeXt and EfficientNet V2 are similar and can reach over 90% accuracy. ShuffleNet V2 has minimal parameters and decent accuracy compared to the rest of the network. By comparison, although SE-ConvNeXt has more parameters than most networks, SE-ConvNeXt is far more accurate than its counterparts.

**Table 4:** Comparison of training parameters and accuracy of each model.

Models	Input size	Output size	Total parameters	Trainable parameters	Accuracy (%)
VGG-16	224	16	70,305,606	70,305,606	84.8
Efficient V2	224	16	20,339,046	20,185,174	92.4
ShuffleNet V2	224	16	1,275,934	1,275,934	93.9
ConvNeXt	224	16	27,824,742	27,824,742	94.6
SE-ConvNeXt	224	16	29,450,430	29,450,430	97.0

Comparing the training process of SE-ConvNex with other convolutional neural networks shows that the SE-ConvNeXt network performs better than other networks in the training process. However, the accuracy on the test set was only 87.99%, significantly different from the 97.3% accuracy during training. Therefore, it cannot be shown by the training process alone that SE-ConvNeXt outperforms other convolutional neural networks. To be able to explore their performance accurately and comprehensively, this paper examines several network models using test sets and validates them with three evaluation metrics.

**Figure 9:**
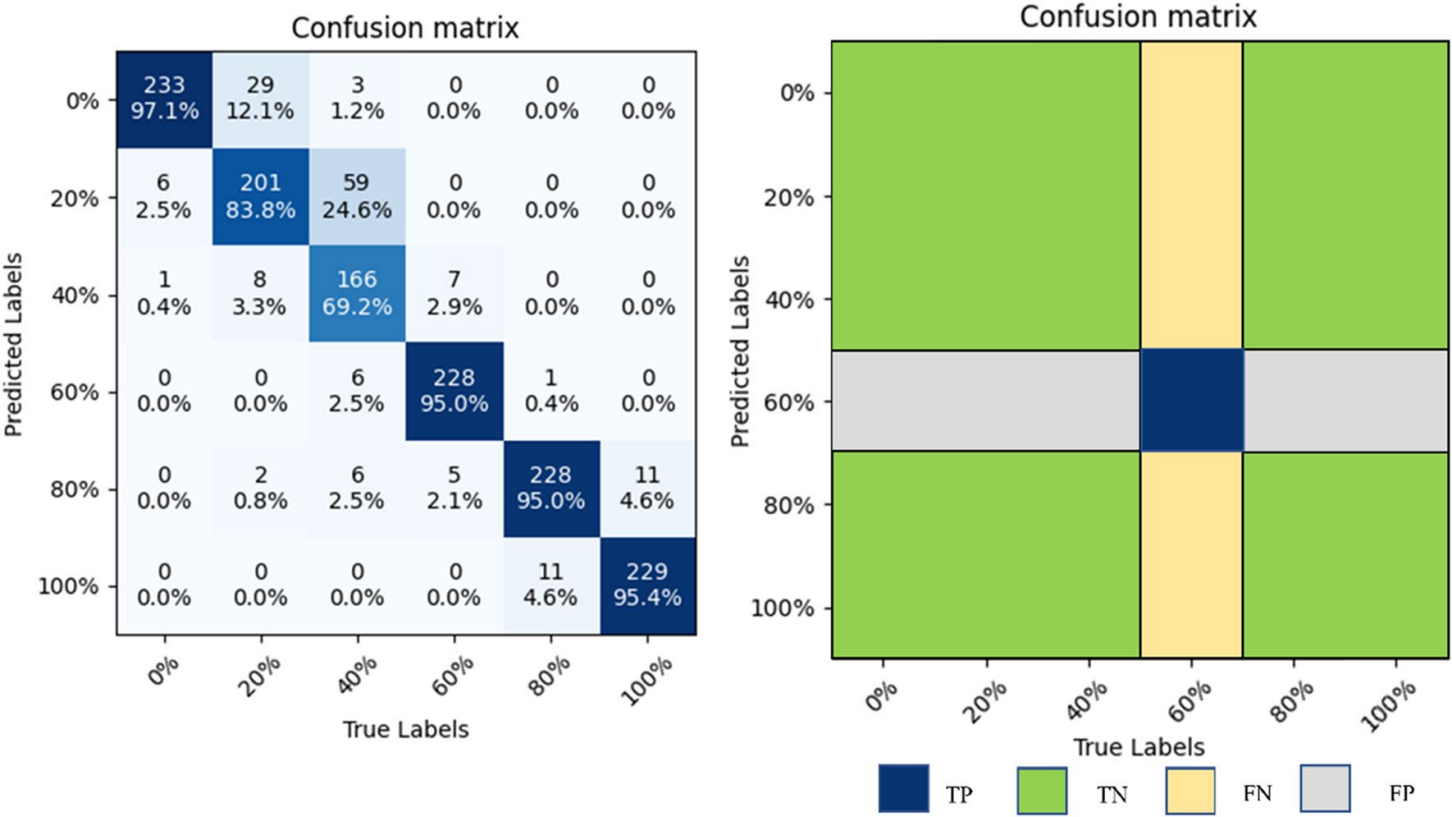
TP, TN, FP, FN calculation example diagram.

The values of TP, TN, FP, and FN need to be obtained to calculate the evaluation index. True Positive (TP): the sample is judged to be positive and is, in fact, positive. True Negative (TN): the sample is judged to be negative and is, in fact, harmful. False Positive (FP): the sample judged to be positive is, in fact, a negative sample. False Negative (FN): the sample judged to be positive is, in fact, harmful. The SE-ConvNeXt confusion matrix is used as an example to calculate TP, TN, FP, and FN for 60% substitution, as shown in Fig. 9. TP = 228, TN = 1193, FN = 12, and FP = 7 for 60% substitution.

The three-evaluation metrics are precision, recall, and F1-score. Precision: in all samples with optimistic predictions. The probability that the sample is positive, as shown in Formula 1. Recall the probability of being predicted as a positive sample out of an actual positive sample, shown in Eq. (2). F1-score: the harmonic mean of precision rate and recall rate, shown in Eq. (3).

1 $$\begin{array}{c}{{P}}{{r}}{{e}}{{c}}{{i}}{{s}}{{i}}{{o}}{{n}}{=}\frac{{TP}}{{TP+FP}}\end{array}$$

2 $$\begin{array}{c}{{R}}{{e}}{{c}}{{a}}{{l}}{{l}}{=}\frac{{TP}}{{TP+FN}}\end{array}$$

3 $$\begin{array}{c}{{F}}_{{\text{1-}}{{score}}}{=}\frac{1}{\frac{1}{{{Precision}}}{+}\frac{1}{{{Recall}}}}\end{array}$$

This paper uses accuracy, precision, recall, and F1 index to compare further four models, SE-ConvNeXt, Efficient V2, ShuffleNet V2, and VGG-16. The accuracy of the four models on the test set was 87.99, 52.97, 82.99, and 82.99%, respectively. The accuracy rate has decreased compared to the accuracy rate during training. However, the accuracy of the SE-ConvNeXt model is still better than the remaining three networks.

**Figure 10:**
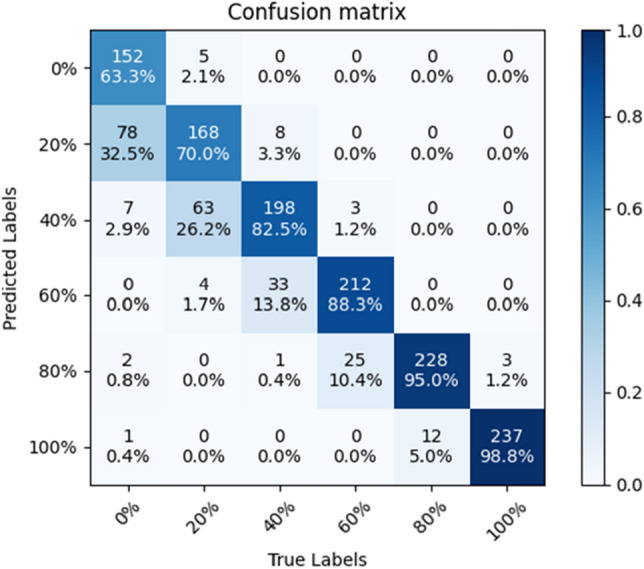
Confusion matrix of ShuffleNet V2.

**Figure 11:**
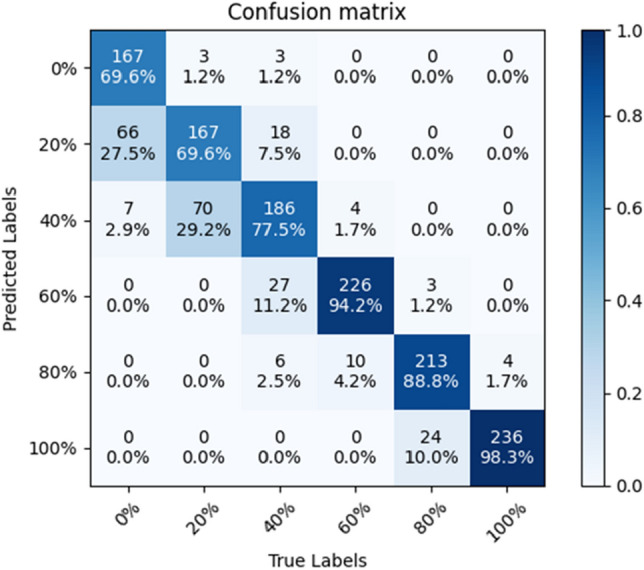
Confusion matrix of VGG-16.

The precision, recall, and F1 index of each model at each substitution rate are shown in Table 5. The confusion matrix of ShuffleNet V2 is shown in Fig. 10. The confusion matrix for VGG-16 is shown in Fig. 11. The accuracy of EfficientNet V2 is lower and will not be compared. Table 5 and the confusion matrix shows that the three network models are generally less accurate when predicting lower substitution rates. The confused image is mainly in the range of adjacent substitution rates. It is consistent with the change rule of the mixed sand sample.

**Table 5:** Precision, recall, F1-score for each substitution rate. This is a note. M1 is the SE-ConvNeXt model, M2 is the ShuffleNet V2 model, and M3 is the VGG-16 model.

Indicators		Precision			Recall			F1-score		
		M 1 (%)	M 2 (%)	M 3 (%)	M 1 (%)	M 2 (%)	M 3 (%)	M 1 (%)	M 2 (%)	M 3 (%)
Substitution rate	0%	87.9	96.8	96.5	97.1	63.3	69.6	92.3	76.5	80.9
	20%	75.6	66.1	66.5	83.8	70.0	69.6	79.5	68.0	68.0
	40%	91.2	73.1	69.7	69.2	82.5	77.5	78.7	77.5	73.4
	60%	97.0	85.1	88.3	95.0	88.3	94.2	96.0	86.7	91.2
	80%	90.5	88.0	91.4	95.0	95.0	88.8	92.7	91.4	90.1
	100%	95.4	94.8	90.8	95.4	98.8	98.3	95.4	96.8	94.4

Precision measures the accuracy of the model's positive predictions, with a higher precision indicating a lower false positive rate when predicting positive instances. For SE-ConvNeXt, the precision rates for predicting 40, 60, 80, and 100% are 91.2, 97.0, 90.5, and 95.4%, respectively, showing high precision in predicting these replacement rates. When expecting a 20% replacement rate, SE-ConvNeXt achieves a precision rate of 75.6%, which is 9.5% and 9.1% higher than that of ShuffleNet V2 and VGG-16 models, respectively, with a relatively lower false positive rate. When predicting a 0% replacement rate, SE-ConvNeXt achieves a precision rate of 87.9%, lower than ShuffleNet V2 and VGG-16 but still relatively high.

Recall measures the coverage of positive class samples by the model, with a higher recall indicating a more vital ability to predict positive instances. For SE-ConvNeXt, the recall rates for predicting 0, 60, 80, and 100% replacement rates are 97.1, 95.0, 95.0, and 95.4%, respectively, indicating high recall rates for these replacement rates. When predicting a 20% replacement rate, SE-ConvNeXt achieves a recall rate of 83.8%, which is 13.8% and 14.2% higher than that of ShuffleNet V2 and VGG-16, respectively, showing a relatively high recognition capability. However, when predicting a 40% replacement rate, the recall rate of SE-ConvNeXt is lower than ShuffleNet V2 and VGG-16, indicating a lower recognition ability.

Precision and recall are competing metrics when evaluating model performance. Improving precision may lead to a decrease in recall, and vice versa. Precision only provides accurate information about positive predictions while ignoring samples incorrectly classified as positive. Recall focuses on the model's ability to identify positive instances while ignoring errors in classifying negative instances. The F1 score combines both metrics to provide a more comprehensive model performance evaluation. For SE-ConvNeXt, the F1 scores for predicting 0%, 60%, 80%, and 100% replacement rates are 92.3, 96.0, 92.7, and 95.4%, respectively, indicating good performance. When predicting a 20% replacement rate, the F1 score for SE-ConvNeXt is 79.5%, 11.5% higher than that of ShuffleNet V2 and VGG-16. For predicting a 40% replacement rate, the F1 score for SE-ConvNeXt is 78.7, 1.5, and 5.3% higher than that of ShuffleNet V2 and VGG-16, respectively. By comparing the F1 scores, SE-ConvNeXt demonstrates the best overall performance, particularly in predicting steel slag replacement rates, showing significant advantages.

### Comparative experiment of transfer learning

Transfer learning has the advantages of shortening training time, reducing model training cost, and achieving ideal results with smaller data sets^35^. The essence of transfer learning is to transfer knowledge to other fields and reuse it. The weights and parameters of pre-training are loaded into the new model to help the network learn features from experience.

**Figure 12:**
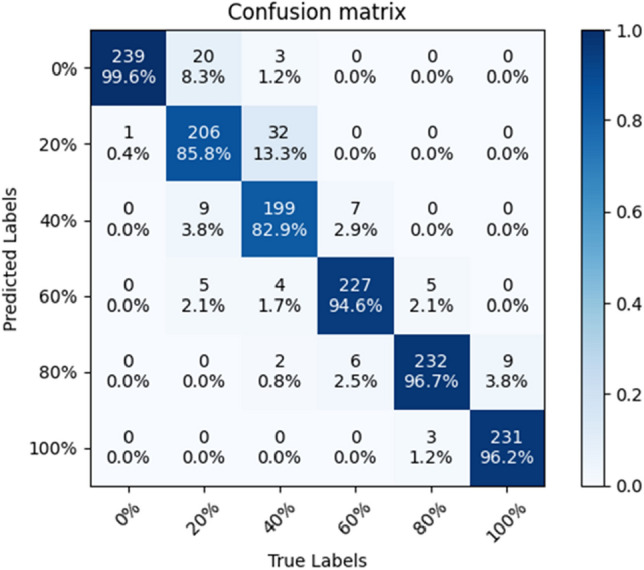
Confusion matrix of SE-ConvNeXt with transfer learning.

We failed to find tasks similar to the classification in this paper on the network. Therefore, the model is pre-trained by selecting a set of mixed sand data sets. In order to obtain optimal experimental results, all parameters of the model are trained in transfer learning. Table 6 shows the F1-score comparison of SE-ConvNeXt before and after using the transfer learning approach, and Fig. 12 shows the confusion matrix of the SE-ConvNeXt with transfer learning. The SE-ConvNeXt with transfer learning, the model achieved an accuracy of 92.64% in the test set. The results shown in Table 6 and Fig. 12 shows that the F1-score of almost all substitution rates has increased. The number of images confused by the model is reduced, and the model's performance is improved considerably.

**Table 6:** F1-score metrics for SE-ConvNeXt with transfer learning.

	0%	20%	40%	60%	80%	100%
With transfer learning	0.923	0.795	0.787	0.960	0.927	0.954
Without transfer learning	0.952	0.860	0.875	0.944	0.949	0.974

During pre-training, the model learned some general features, such as color, from mixed sand samples. These features can be transferred to the model that recognizes the three groups of mixed sands, helping it better learn the characteristics of the three types of mixed sands. Moreover, the model that identifies a kind of mixed sand replacement rate shares similarities with the model that recognizes the replacement rates of the three types of mixed sand. Therefore, the feature representation capability learned by the pre-trained model is more easily adaptable to the requirements of recognizing the three groups of mixed sands, thereby improving the accuracy and F1 score of the model.

**Figure 13:**
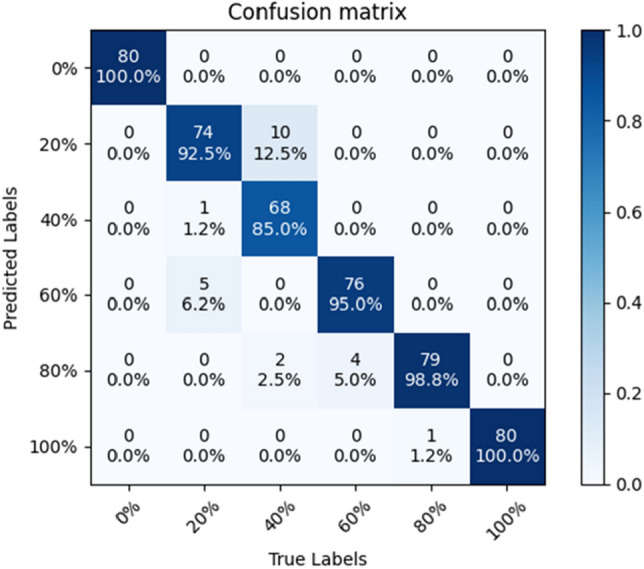
Confusion matrix of the SE-ConvNeXt with transfer learning predicting the B-sample test set.

In this paper, we use the model with transfer learning to predict the test set of B and C samples, respectively, and compare it with the model without transfer learning. The model with transfer learning can predict the B-sample test set with an accuracy of 95.21%, and the confusion matrix is shown in Fig. 13. With the model predicting a 40% substitution rate, the accuracy of this model, which is 85%, increases by 36.25% compared with the model without transfer learning. The accuracy of predicting sand blends with more than 60% substitution was 97.9%, and the model predicted sand blends with more than 60% substitution with high accuracy. In the model with transfer learning, the model can better extract the features of the B-sample, and the accuracy of predicting the B-sample is improved significantly.

**Figure 14:**
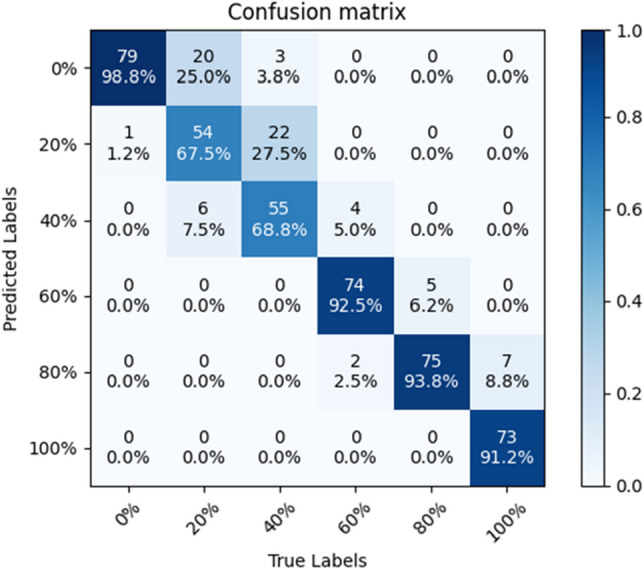
Confusion matrix of the SE-ConvNeXt with transfer learning predicting the C-sample test set.

The model with transfer learning can predict the C-sample test set with an accuracy of 85.42%, and the confusion matrix is shown in Fig. 14. The model's accuracy in predicting 20% substitution was 67.5%, and the accuracy in predicting 40% substitution was 68.75%, a slight improvement compared to the model without transfer learning. The accuracy of predicting sand blends with more than 60% substitution was 92.5%, and the model predicted sand blends with more than 60% substitution with high accuracy. The SE-ConvNeXt model accurately identified the substitution rate of steel slag sand in A and B sand mixes. However, the improvement in accuracy was less in determining C mixed sand. In particular, the improvement in accuracy was only 8.7 and 3.8% when determining 20 and 40% substitution rates, respectively. In type C blended sand, when the substitution rate of steel slag sand is low, the 20 and 40% blended sand are similar to the combined sand with a lower substitution rate, and the difference is minimal. Even using migration learning, these subtle differences are difficult to be accurately captured by the model, resulting in limited improvement in accuracy. However, compared with the model accuracy without transfer learning, there is a significant improvement.

## Conclusion

For the quality control problem of steel slag sand substitution rate, the image recognition method is used to quickly detect the steel slag sand substitution rate and achieve the proper application of steel slag sand in the project. In this paper, the ConvNeXt network is used as the main framework. The SE-ConvNeXt network model is architected by integrating the SE attention mechanism module in the module of ConvNeXt for the color characteristics of mixed sand. They are aiming at the problem of low prediction accuracy of the model under a low substitution rate. The transfer learning training method is used to train all the parameters in the model to improve accuracy of the model.

1. Through the five cross-validation methods of SE-ConvNeXt, the model predicts that the accuracy of the steel slag mixed sand test set is 87.99%. The accuracy of predicting A, B, and C samples was 96.88, 88.54, and 82.29%, respectively. Show the excellent performance of the model.
2. Comparing SE-ConvNeXt and ConvNeXt and analyzing the effect of the SE attention mechanism module on ConvNeXt, the training process of the ConvNeXt model after adding SE is more stable, with better convergence and higher accuracy.
3. SE-ConvNeXt was compared with five convolutional neural network models under the same steel slag sand dataset condition with confusion matrix and evaluation metrics.SE-ConvNeXt has the highest accuracy with the addition of fewer training parameters.
4. The SE-ConvNeXt with transfer learning achieved 92.64% accuracy in predicting the three sand blends, an improvement of 4.65%.

The above research results show that SE-ConvNeXt can obtain higher accuracy in the classification task of steel slag sand substitution rate through the training method of transfer learning. It can quickly and accurately identify the substitution rate of steel slag sand.

## Data Availability

The datasets used and/or analysed during the current study available from the corresponding author on reasonable request.

## References

[1] Venkatesan B, Lijina VJ, Kannan V, Dhevasenaa PR (2021). Partial replacement of fine aggregate by steel slag and coarse aggregate by walnut shell in concrete. Mater. Today Proc..

[2] Dong Q, Wang G, Chen X, Tan J, Gu X (2020). Recycling of steel slag aggregate in portland cement concrete: An overview. J. Clean. Prod..

[3] Gencel O, Karadag O, Oren OH, Bilir T (2021). Steel slag and its applications in cement and concrete technology: A review. Constr. Build. Mater..

[4] Li Z, Shen A, Yang X, Guo Y, Liu Y (2022). A review of steel slag as a substitute for natural aggregate applied to cement concrete. Road Mater. Pavement Des..

[5] Chen X, Wang G, Dong Q, Zhao X, Wang Y (2020). Microscopic characterizations of pervious concrete using recycled steel slag aggregate. J. Clean. Prod..

[6] Rashad AM (2022). Behavior of steel slag aggregate in mortar and concrete—a comprehensive overview. J. Build. Eng..

[7] Jiang F, Liu X (2020). Experimental study of steel slag-fly ash ready-mixed mortar. New Build. Mater..

[8] Rehman S, Iqbal S, Ali A (2018). Combined influence of glass powder and granular steel slag on freshand mechanical properties of self-compacting concrete. Constr. Build. Mater..

[9] Pan S, Chen D, Chen X, Ge G, Liu C (2020). Experimental study on the workability and stability of steel slag self-compacting concrete. Appl. Sci..

[10] Jin Q, Mao F, Zhao X, Su Z (2022). Comparative study on influence of different replacement methods of steel slag sand on workability and mechanical properties of dry-mixed mortar. J. Xinjiang Agric. Univ..

[11] Ma K (2021). The morphological characteristics of brick-concrete recycled coarse aggregate based on the digital image processing technique. J. Build. Eng..

[12] Han J, Wang K, Wang X, Monteiro PJM (2016). 2D image analysis method for evaluating coarse aggregate characteristic and distribution in concrete. Constr. Build. Mater..

[13] Cao Y, Yang G, Zhang Y, Wang R, Cheng Z (2019). Rapid evaluation method of shape characteristics of aggregate particle based on the minimum outer rectangle. J. Chongqing Jiaotong Univ. (Nat. Sci. Ed.).

[14] Krizhevsky A, Sutskever I, Hinton GE (2012). ImageNet classification with deep convolutional neural networks. Adv. Neural. Inf. Process. Syst..

[15] Simonyan, K. & Zisserman, A. Very deep convolutional networks for large-scale image recognition. Preprint at https://arxiv.53yu.com/abs/1409.1556 (2014).

[16] He, K., Zhang, X., Ren, S. & Sun, J. Deep residual learning for image recognition. In *2016 IEEE Conference on Computer Vision and Pattern Recognition (CVPR)* Las Vegas, NV, USA, 770–778 (2016).

[17] Howard, A. G. *et al.* MobileNets: Efficient convolutional neural networks for mobile vision applications. Preprint at https://arxiv.53yu.com/abs/1704.04861 (2017).

[18] Zhang, X., Zhou, X., Lin, M. & Sun, J. ShuffleNet: An extremely efficient convolutional neural network for mobile devices. In *2018 IEEE/CVF Conference on Computer Vision and Pattern Recognition*. Salt Lake City, UT, USA, 6848–6856 (2018).

[19] Tan, M. & Le, Q. V. EfficientNet: Rethinking model scaling for convolutional neural networks. In *Proceedings of the 36th International Conference on Machine Learning*. PMLR 139, 6105–6114 (2019).

[20] Dan HC, Bai GW, Zhu ZH (2021). Application of deep learning-based image recognition technology to asphalt–aggregate mixtures: Methodology. Constr. Build. Mater..

[21] Hoong J, Lux J, Mahieux PY, Turcry P, At-Mokhtar A (2020). Determination of the composition of recycled aggregates using a deep learning-based image analysis. Autom. Constr..

[22] Hoong, J., Lux, J., Mahieux, P. Y., Turcry, P., & At-Mokhtar, A. classification of recycled aggregates using deep learning. In* Proceedings of the 3rd RILEM Spring Convention and Conference (RSCC 2020)* Volume 4: Shift to a Circular Economy 3, 21–32 (2020).

[23] Su C, Zhang H, Wang W (2022). Automatic segmentation of concrete aggregate using convolutional neural network. Autom. Constr..

[24] Hu, J., Shen, L. & Sun, G. Squeeze-and-excitation networks. In *2018 IEEE/CVF Conference on Computer Vision and Pattern Recognition*. Salt Lake City, UT, USA, 7132–7141 (2018).

[25] Liu, Z., Mao, H., Wu, C. Y. Feichtenhofer, C., Darrell, T., & Xie, S. A convnet for the 2020s. In *2021 IEEE/CVF International Conference on Computer Vision (ICCV)*. New Orleans, LA, USA, 11966–11976 (2022).

[26] Oquab, M., Bottou, L., Laptev, I. & Sivic, J. Learning and transferring mid-level image representations using convolutional neural networks project page. In *Proceedings of the IEEE Conference on Computer Vision and Pattern Recognition*. Columbus, OH, USA, 1717–1724 (2014).

[27] Liu, Z., Lin, Y., Cao, Y., Hu, H., Wei, Y. & Zhang, Z. Swin transformer: hierarchical vision transformer using shifted windows. In *2022 IEEE/CVF Conference on Computer Vision and Pattern Recognition (CVPR)*. Montreal, QC, Canada, 9992–10002 (2021).

[28] Touvron, H., Cord, M., Sablayrolles, A., Synnaeve, G. & Jégou, H. Going deeper with image transformers. In *2021 IEEE/CVF International Conference on Computer Vision (ICCV)*. Montreal, QC, Canada, 32–42 (2021).

[29] Larsson, G., Maire, M. & Shakhnarovich, G. FractalNet: Ultra-deep neural networks without residuals. Preprint at https://arxiv.53yu.com/abs/1605.07648 (2016).

[30] Hendrycks, D. & Gimpel, K. Gaussian error linear units (GELUs). Preprint at https://arxiv.53yu.com/abs/1606.08415 (2016).

[31] Nair, V. & Hinton, G. E. Rectified linear units improve restricted boltzmann machines. In *Proceedings of the 27th International Conference on Machine Learning (ICML-10)*. Haifa, Israel, 807–814 (2010).

[32] Tan M, Le QV (2021). EfficientNetV2: Smaller models and faster training. Int. Conf. Mach. Learn..

[33] Howard, A. et al. Searching for MobileNetV3. In *2019 IEEE/CVF International Conference on Computer Vision (ICCV)*. Seoul, Korea (South), 2019, 1314–1324 (2019).

[34] Ma, N., Zhang, X. & Zheng, H. T. ShuffleNet V2: Practical guidelines for efficient CNN. In *2018 European Conference on Computer Vision (ECCV)* 116–131 (Springer, Heidelberg, 2018).

[35] Dyson J, Mancini A, Frontoni E, Zingaretti P (2019). Deep learning for soil and crop segmentation from remotely sensed data. Remote Sens..

